# Impact of intertrochanteric bone mineral density on the accuracy of preoperative templating in cementless total hip arthroplasty: a retrospective cohort study of 510 hips

**DOI:** 10.1186/s12891-026-10301-5

**Published:** 2026-08-04

**Authors:** Min Uk Do, Kyeong Baek Kim, Sang-Min Lee, Hyun Tae Koo, Kuen Tak Suh, Won Chul Shin

**Affiliations:** 1 Department of Orthopedic Surgery, Research Institute for Convergence of Biomedical Science and Technology, Pusan National University Yangsan Hospital, Pusan National University School of Medicine, 20 Geumo-ro, Mulgeum-eup, Yangsan, Gyeongsangnam-do 626-770 Republic of Korea; 2 Department of Orthopedic Surgery, Mirae Hospital, Pusan National University School of Medicine, Busan, Republic of Korea

**Keywords:** Total hip arthroplasty, Preoperative templating, Bone mineral density

## Abstract

**Background:**

Preoperative templating accuracy in cementless total hip arthroplasty (THA) may be influenced by patient-specific and radiographic factors, yet bone quality has rarely been considered. We hypothesized that bone mineral density (BMD), and particularly intertrochanteric BMD, would be associated with femoral stem size discrepancy.

**Methods:**

In a retrospective cohort of 510 hips (467 patients) undergoing primary cementless THA, we compared templated and actual implant sizes for both femoral and acetabular components and assessed clinical and radiographic variables, including age, sex, weight, body mass index, BMD (T-score) at multiple proximal femoral sites, Dorr classification, and surgeon training level, using univariate and multivariable linear regression. Receiver operating characteristic analysis evaluated the discriminative performance of BMD for substantial stem size discrepancies.

**Results:**

No factor was associated with acetabular cup size discrepancy. Age, weight, and intertrochanteric BMD were independently associated with femoral stem size discrepancy, with intertrochanteric BMD showing the strongest association among the variables examined (standardized β = −0.326). A T-score cutoff of − 0.9 was associated with discrepancies of at least three sizes larger than templated (area under the curve 0.782), and a cutoff of 0.2 with discrepancies of at least three sizes smaller (area under the curve 0.825).

**Conclusions:**

Among the factors examined, intertrochanteric BMD showed the strongest association with femoral stem size discrepancy, although it explained only part of the overall variability. Preoperative assessment of intertrochanteric BMD may help anticipate substantial deviations in stem sizing and ensure that appropriately sized implants are available during surgery.

**Supplementary Information:**

The online version contains supplementary material available at https://doi.org/10.1186/s12891-026-10301-5.

## Background

The importance of preoperative templating in total hip arthroplasty (THA) is well established. Templating facilitates the optimization of postoperative offset and correction of leg length discrepancy (LLD) and also enables accurate prediction of appropriate acetabular cup and femoral stem sizes [1–4]. Effective preoperative templating not only enhances surgical efficiency by reducing operative time but also helps prevent complications [5, 6]. Several studies have sought to improve templating accuracy by incorporating patient-specific characteristics, such as age, height, weight, and body mass index (BMI). However, these investigations did not consider bone quality [7–9].

In cementless THA, initial implant stability is achieved through press-fitting, which relies on cortical bone contact [10]. Consequently, bone quality, reflected in both Dorr classification and bone mineral density (BMD), is a critical factor associated with initial fixation [11]. Indeed, prior studies have reported suboptimal outcomes in patients with low BMD, including poor initial fixation and an increased risk of implant migration [12–14]. We therefore hypothesized that bone quantity, reflected by DXA-derived BMD, would influence femoral stem size discrepancy, such that lower BMD would be associated with the use of larger implants compared to the templated size. In particular, we hypothesized that intertrochanteric BMD, which reflects proximal femoral bone supporting press-fit fixation, would be most strongly associated with stem size discrepancy.

Based on this hypothesis, this study was designed to address the following questions:

1. Are patient-related factors, including BMD, associated with implant size discrepancy in THA?
2. Can intertrochanteric BMD identify patients at higher likelihood of clinically relevant discrepancies in femoral stem sizing?

## Methods

This study was approved by the Institutional Review Board of Pusan National University Yangsan Hospital (no. 55-2025-087), and all methods were performed in accordance with the relevant guidelines and regulations, including the Declaration of Helsinki. Because of the retrospective design, the requirement for informed consent was waived by the Institutional Review Board. The study is reported in accordance with the STROBE guidelines.

**Fig. 1:**
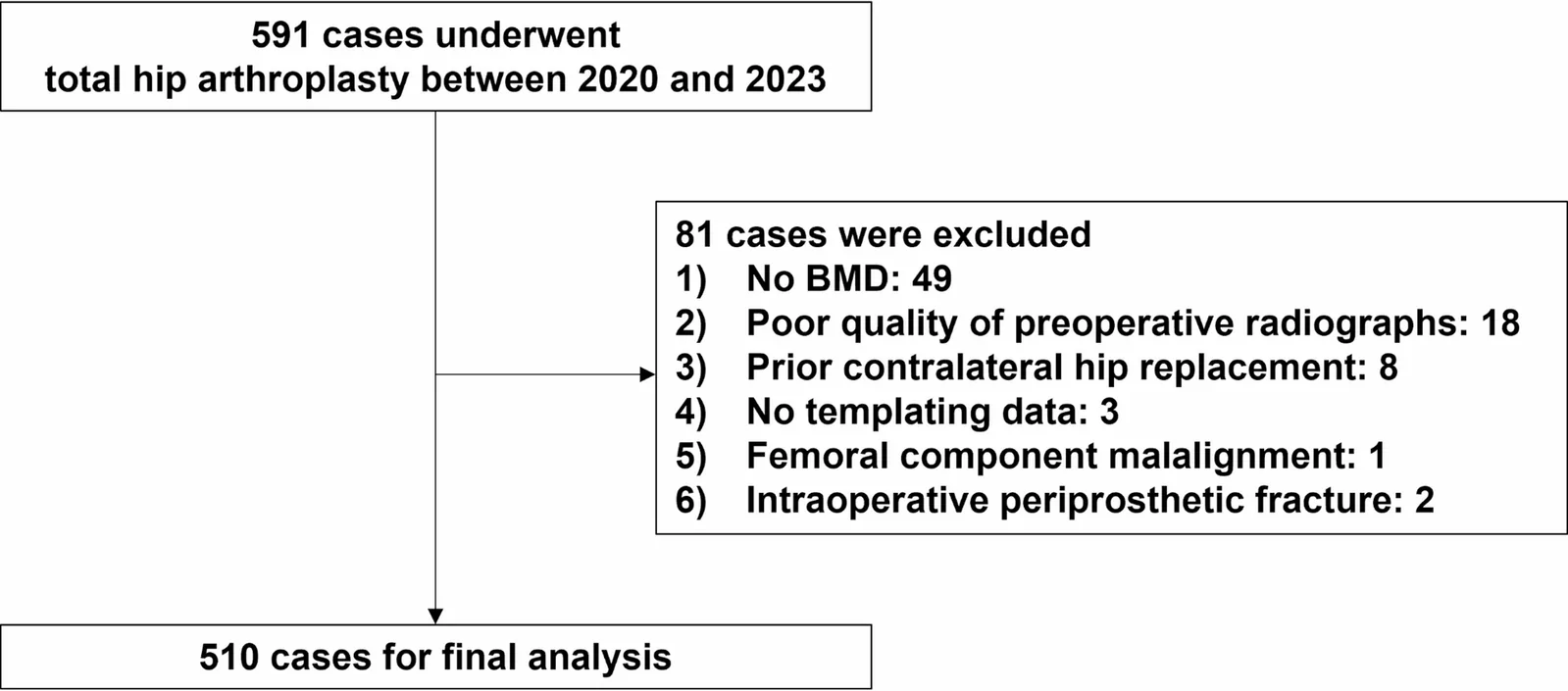
Study flowchart

We conducted a single-institution, retrospective cohort study of patients who underwent primary THA between March 2020 and April 2023. A total of 591 cases were initially identified using the electronic medical record system. Of these, 49 cases were excluded because DXA had not been performed within three months before surgery, and 18 were excluded because poor-quality preoperative radiographs prevented accurate templating. Eight cases were excluded because the patients had previously undergone contralateral THA, for which the prior implant size was already known. Three cases were excluded because of missing templating records, and one case was excluded because of stem malalignment exceeding 5° of varus or valgus. In addition, two cases with intraoperative periprosthetic fracture were excluded because the fracture was considered to be related to substantial stem size mismatch and could directly affect the final implanted stem size, thereby confounding the assessment of templating accuracy. No postoperative periprosthetic fractures were observed. After applying these criteria, 510 hips in 467 patients were included in the final analysis (Fig. 1). Forty-three patients underwent simultaneous same-day bilateral THA. Each hip was templated independently and analyzed as a separate observation, and a single preoperative DXA scan performed within three months before surgery was used for both hips.

Collected data included age, sex, height, weight, BMI, BMD (T-score, measured at the femoral neck, trochanteric region, intertrochanteric region, and total hip), Dorr classification, diagnosis leading to THA, femoral and acetabular components used, surgeon’s training level (postgraduate year) at the time of templating, postoperative LLD, femoral offset difference, and implant size discrepancy. BMD was assessed using DXA with a Horizon W scanner (Hologic, Marlborough, MA, USA). Preoperative DXA was routinely performed as part of standard perioperative care before primary THA. Although patients with osteoporosis or low BMD were managed according to institutional practice, treatment was generally initiated after surgery. Therefore, osteoporosis management was unlikely to influence surgical planning, intraoperative decision-making, or final stem size selection. Implant size discrepancy was defined as the difference between the actual implant size used during surgery and the preoperatively templated size, with positive values indicating the use of a larger implant than planned. This discrepancy was calculated separately for the femoral stem (stem size discrepancy) and the acetabular cup (cup size discrepancy); throughout the manuscript, “stem size discrepancy” refers specifically to the femoral component, whereas “implant size discrepancy” is used only when referring collectively to both components. The mean patient age was 56.9 years. Of the included cases, 235 (46.1%) were female and 275 (53.9%) were male. The mean BMI was 24.9 kg/m². Mean T-scores were 0.6 (femoral neck), 0.7 (trochanteric), 0.6 (intertrochanteric), and 0.6 (total hip). By World Health Organization criteria, 300 hips (58.8%) were normal, 172 (33.7%) osteopenic, and 38 (7.5%) osteoporotic. Dorr classification was independently assessed by two orthopedic surgeons (MUD and WCS), yielding 185 type A (36.3%), 298 type B (58.4%), and 27 type C (5.3%) hips. Inter-observer reliability for the Dorr classification was substantial (κ = 0.780). The most common diagnosis was femoral head osteonecrosis (315 cases, 61.8%), followed by osteoarthritis (179 cases, 35.1%), and other conditions (3.1%). LLD was assessed on postoperative anteroposterior radiographs by drawing a line across the inferior borders of both ischial tuberosities and measuring the vertical distance to the most superior aspect of each lesser trochanter [15]. Based on previous literature, an LLD ≤ 5 mm was considered optimal, while an LLD > 10 mm was considered clinically significant [16–18]. The mean postoperative LLD was 1.1 mm, indicating accurate restoration of leg length. LLD ≤ 5 mm was observed in 475 cases (93.1%), while no patients had an LLD greater than 10 mm. Femoral offset was measured using the Sundsvall method, defined as the horizontal distance between the pelvic midline and the femoral axis at the level of the most lateral border of the greater trochanter [19]. The mean difference in femoral offset was 1.5 mm (Table 1).

**Table 1:** Preoperative demographics

Parameter	Total
Number	510
Age, mean ± SD, years	56.9 ± 14.3
Sex	
Female	235 (46.1%)
Male	275 (53.9%)
BMI, mean ± SD, kg/m^2^	24.9 ± 4.0
BMD, mean ± SD, T-score (femoral neck)	0.6 ± 1.4
BMD, mean ± SD, T-score (trochanteric)	0.7 ± 1.0
BMD, mean ± SD, T-score (intertrochanteric)	0.6 ± 1.1
BMD, mean ± SD, T-score (total hip)	0.6 ± 1.1
Dorr classification	
A	185 (36.3%)
B	298 (58.4%)
C	27 (5.3%)
Cause for THA	
ONFH	315 (61.8%)
OA	179 (35.1%)
FNF	9 (1.8%)
SIF	3 (0.6%)
Post-traumatic OA	2 (0.4%)
AS	2 (0.4%)
Femoral component	
Taperloc Microplasty (Zimmer Biomet)	510 (100.0%)
Acetabular component	
G7 (Zimmer Biomet)	510 (100.0%)
Templating surgeon training level	
PGY-2	103 (20.2%)
PGY-3	87 (17.1%)
PGY-4	79 (15.5%)
PGY-5	112 (22.0%)
PGY-6	129 (25.3%)
Postoperative LLD	1.1 ± 2.3
LLD > 5 mm	35 (6.9%)
LLD > 10 mm	0 (0.0%)
Difference of femoral offset, mm	1.5 ± 3.2

*SD* standard deviation, *BMI* body mass index, *BMD* bone mineral density, *THA* total hip arthroplasty, *ONFH* osteonecrosis of the femoral head, *OA* osteoarthritis, *FNF* femoral neck fracture, *SIF* subchondral insufficiency fracture, *AS* ankylosing spondylitis, *PGY* postgraduate year, *LLD* leg length discrepancy

All surgeries were performed by an experienced arthroplasty surgeon using the posterolateral approach. Serial broaching of the proximal femur was conducted to determine the optimal implant size by aligning the broach with the native femoral version. Final stem size was determined intraoperatively by sequential broaching and tactile assessment of press-fit; routine intraoperative fluoroscopy or radiography was not used. The femoral component used in all cases was the Taperloc Microplasty (Zimmer Biomet, Warsaw, IN, USA; *n* = 510, 100%), and the acetabular component was the G7 system (Zimmer Biomet; *n* = 510, 100%).

**Fig. 2:**
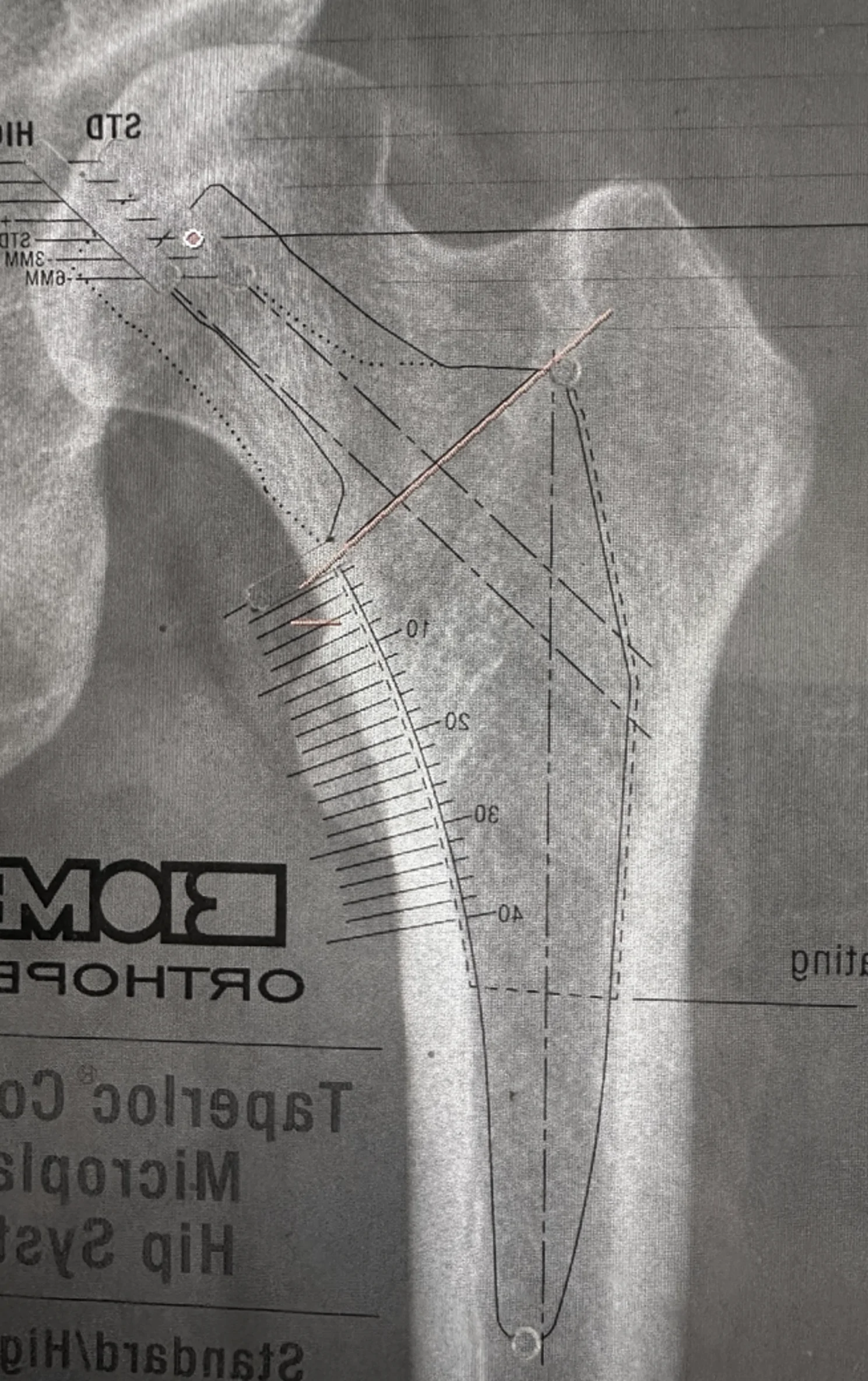
Acetate templating on a digital radiograph to estimate the anticipated femoral component size

Preoperative templating was performed using calibrated digital radiographs on the picture archiving and communication system (PACS) (INFINITT PACS M6 version; INFINITT Healthcare, Seoul, Korea), with acetate templates displayed on the monitor. The same PACS version and workflow were used throughout the study period without change. Anteroposterior (AP) pelvic radiographs were obtained with both femurs internally rotated by 15°–20°, and lateral views were additionally acquired. A 10-cm radiopaque calibration bar was placed adjacent to the pelvis for AP views and on the anterior thigh for lateral views. Radiographic magnification was adjusted individually by matching the projected length of the calibration bar to its actual length using the PACS scaling function [20]. Acetabular cup size and positioning were determined using both AP and lateral radiographs. The template was aligned at an inclination angle of 40°–45°, positioned lateral to the teardrop and above the obturator foramen. Femoral templating was performed according to a standardized planning concept for a proximally coated, flat tapered-wedge stem with a porous plasma-sprayed surface. The aim was to select the smallest stem size that provided adequate proximal metaphyseal fill while avoiding excessive diaphyseal cortical engagement. The stem template was aligned along the femoral canal axis, with intended medial contact at and immediately below the calcar and lateral contact at the metaphyseal flare. The final templated stem size was defined as the size that best filled the metaphysis while restoring the planned center of rotation, femoral offset, and leg length. The standard 133° neck-shaft angle option of the Taperloc Microplasty was used in most cases, while the high-offset 133° option was reserved for cases requiring additional femoral offset restoration. Femoral head and neck length were assumed to be the standard 0-mm option during templating (Fig. 2). Templating was primarily performed by orthopedic residents or clinical fellows and categorized according to their postgraduate year (PGY). All trainees applied a single standardized templating protocol under the supervision of the operating surgeon, and every plan was reviewed and, when necessary, revised by the operating surgeon before surgery. Consequently, the final templated sizes analyzed here reflect a consistent templating philosophy rather than variation between individual trainees. Templating was performed without knowledge of the patients’ BMD measurements.

### Statistical analysis

Continuous variables are presented as means ± standard deviations and categorical variables as frequencies and percentages. Because the implant system uses clinically ordered, approximately sequential size increments, stem size discrepancy was treated as a continuous difference score that retains both the direction and magnitude of deviation. Linear regression was therefore used. In addition, a proportional-odds ordinal logistic regression was performed as a sensitivity analysis and yielded concordant results. Univariate linear regression was used to assess the associations between clinical or radiographic factors (age, sex, height, weight, BMI, BMD, Dorr classification, and training level of the templating surgeon) and discrepancies in stem or cup size. Spearman’s rho (ρ) correlation analysis was performed to evaluate potential multicollinearity. Among variables significantly associated with stem size discrepancy in univariate analyses, those without strong collinearity (|ρ| < 0.7) were selected for multivariable linear regression. Diagnostic heterogeneity was assessed in sensitivity analyses by adding diagnosis (osteonecrosis vs. osteoarthritis vs. other) to the primary multivariable model and by repeating the analysis in the osteonecrosis subgroup. To account for the 43 patients contributing both hips, the multivariable analysis was repeated using a linear mixed-effects model with a random intercept for patient. This mixed-effects model was regarded as a confirmatory analysis; because its estimates were essentially identical to those of the primary model, the simpler linear regression treating each hip as an independent observation is presented as the primary analysis. Receiver operating characteristic (ROC) curve analyses were subsequently conducted to determine the optimal threshold values for significant parameters using the Youden index. A discrepancy of ≥ 3 sizes was selected as the ROC threshold because previous templating studies commonly report femoral stem accuracy within ± 1 or ± 2 sizes, while larger deviations are uncommon and may exceed the range of implants routinely prepared for surgery [21, 22]. Exploratory ROC analyses also showed better discrimination at this threshold than at the one- or two-size levels. All statistical analyses were performed using R version 4.1.3 (R Foundation for Statistical Computing, Vienna, Austria) with RStudio (RStudio, PBC, Boston, MA, USA). A two-tailed *p*-value < 0.05 was considered statistically significant.

## Results

### Templating accuracy

For the femoral component, an exact match between the templated and implanted sizes was achieved in 191 of 510 hips (37.5%). When allowing for a one-size difference, the match rate increased to 396 cases (77.6%), and within two sizes, the accuracy reached 464 cases (91.0%). For the acetabular component, exact matches were observed in 379 cases (74.3%), and 507 cases (99.4%) were within one template size (Table 2).

**Table 2:** Accuracy of preoperative templating of femoral and acetabular components

Parameter	Total	Exact size	± 1 size (%)	± 2 size (%)
Femoral component	510	191 (37.5%)	396 (77.6%)	464 (91.0%)
Acetabular component	510	379 (74.3%)	507 (99.4%)	509 (99.8%)

### Factors associated with implant size discrepancy

No factors were significantly associated with acetabular cup size discrepancy in the univariate linear regression analysis (Table 3). In contrast, several variables were significantly associated with femoral stem size discrepancy, including age (β = 0.026, *p* < 0.001), sex (β = −0.486, *p* < 0.001), height (β = −0.036, *p* < 0.001), weight (β = −0.035, *p* < 0.001), BMI (β = −0.078, *p* < 0.001), BMD at the femoral neck (β = −0.333, *p* < 0.001), trochanteric (β = −0.532, *p* < 0.001), intertrochanteric region (β = −0.566, *p* < 0.001), total hip (β = −0.557, *p* < 0.001), and Dorr classification (β = 0.697 and 1.742, *p* < 0.001) (Table 3). To assess multicollinearity, Spearman’s rho correlation analysis was conducted for variables associated with stem size discrepancy. Variables showing no strong collinearity (|ρ| < 0.7) were selected for multivariable linear regression. Based on this analysis, age, sex, weight, intertrochanteric BMD, and Dorr type were included in the final model (Additional file 1: Table S1). Because the four DXA-derived BMD sites were strongly intercorrelated (|ρ| > 0.7), only one BMD site was entered to avoid multicollinearity; the intertrochanteric site had the strongest univariate association.

**Table 3:** Univariate linear regression analysis of factors associated with implant size discrepancy

Variables	Stem size discrepancy				Acetabular cup size discrepancy			
	β	SE	*P*-value	*R* ^2^	β	SE	*P*-value	*R* ^2^
Age (years)	0.026	0.004	< 0.001	0.068	0.003	0.002	0.089	0.006
Sex (male)				0.028				0.006
Female	Ref	Ref	Ref		Ref	Ref	Ref	
Male	-0.486	0.126	< 0.001		-0.083	0.047	0.075	
Height (cm)	-0.036	0.007	< 0.001	0.057	-0.004	0.002	0.075	0.006
Weight (kg)	-0.035	0.005	< 0.001	0.102	-0.001	0.002	0.585	< 0.001
BMI (kg/m^2^)	-0.078	0.016	< 0.001	0.047	0.004	0.006	0.768	0.001
BMD (T-score, femoral neck)	-0.333	0.042	< 0.001	0.112	0.016	0.016	0.324	0.002
BMD (T-score, trochanteric)	-0.532	0.057	< 0.001	0.145	0.034	0.021	0.114	0.007
BMD (T-score, intertrochanteric)	-0.566	0.052	< 0.001	0.187	0.013	0.021	0.533	< 0.001
BMD (T-score, total hip)	-0.557	0.053	< 0.001	0.181	0.025	0.021	0.243	0.003
Dorr type				0.094				0.011
A	Ref	Ref	Ref		Ref	Ref	Ref	
B	0.697	0.129	< 0.001		0.072	0.049	0.138	
C	1.742	0.283	< 0.001		-0.139	0.108	0.197	
Templating surgeon training level				0.011				0.020
PGY-2	Ref	Ref	Ref		Ref	Ref	Ref	
PGY-3	-0.244	0.209	0.244		0.183	0.076	0.090	
PGY-4	-0.030	0.215	0.889		-0.033	0.078	0.673	
PGY-5	-0.074	0.196	0.708		0.076	0.071	0.288	
PGY-6	-0.375	0.190	0.079		-0.004	0.069	0.954	

*SE* standard error, *BMI* body mass index, *BMD* bone mineral density, *PGY* postgraduate year

### Independent factors associated with stem size discrepancy

**Fig. 3:**
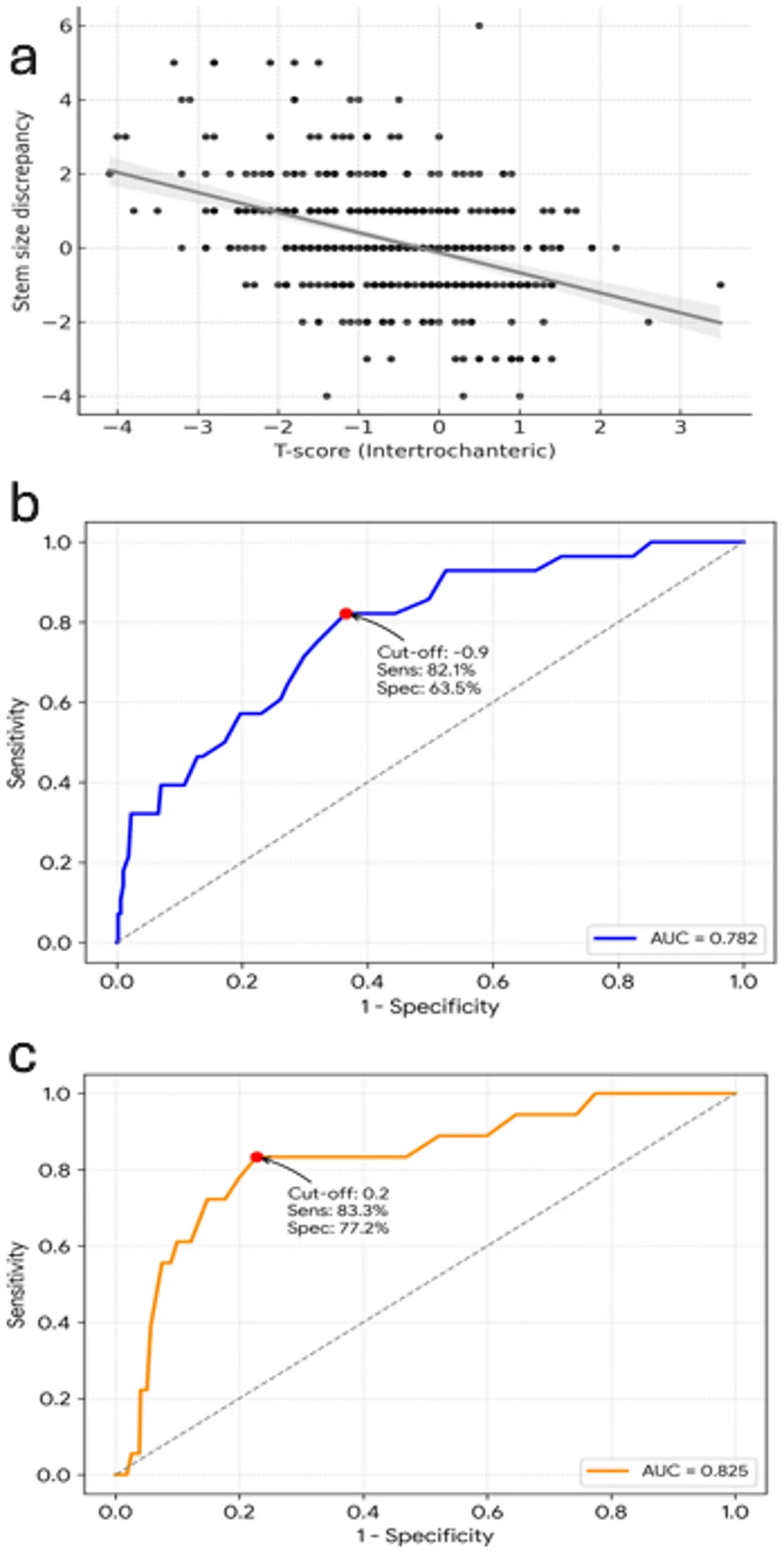
Discriminative performance of intertrochanteric bone mineral density (BMD, T-score) for femoral stem size discrepancy (Df). **a** Scatterplot illustrating the correlation between intertrochanteric BMD and stem size discrepancy. **b** Receiver operating characteristic (ROC) curve for discriminating oversizing (Df ≥ 3). **c** ROC curve for discriminating undersizing (Df ≤ − 3)

In the multivariable model, age (β = 0.013, 95% CI 0.005 to 0.022, *p* = 0.002), weight (β = −0.011, 95% CI − 0.022 to − 0.001, *p* = 0.031), and intertrochanteric BMD (β = −0.427, 95% CI − 0.559 to − 0.294, *p* < 0.001) were independently associated with stem size discrepancy, with intertrochanteric BMD showing the strongest standardized association among the variables examined (standardized β = −0.326) (Fig. 3a; Table 4).

**Table 4:** Multivariable linear regression analysis for factors associated with stem size discrepancy (R^2^ = 0.226)

Variables	β Coefficient	95% CI	Standardized β Coefficient	SE	*P*-value	VIF
Age (years)	0.013	0.005 to 0.022	0.134	0.004	0.002	1.11
Sex (male)						1.15
Female	reference		reference			
Male	-0.032	-0.288 to 0.224	-0.022	0.130	0.807	
Weight (kg)	-0.011	-0.022 to -0.001	-0.108	0.005	0.031	1.27
BMD (T-score, intertrochanteric)	-0.427	-0.559 to -0.294	-0.326	0.067	< 0.001	1.31
Dorr type						1.13
A	reference		reference			
B	0.046	-0.233 to 0.325	0.016	0.142	0.744	
C	0.305	-0.250 to 0.949	0.047	0.305	0.253	

*CI* confidence interval, *SE* standard error, *VIF* variance inflation factor, *BMD* bone mineral density

In sensitivity analyses substituting alternative BMD sites, model fit was similar for total hip (R² = 0.228) and trochanteric (R² = 0.228) and lower for femoral-neck BMD (R² = 0.188). A proportional-odds ordinal model gave a concordant result (intertrochanteric BMD odds ratio 0.516, 95% CI 0.434–0.614, *p* < 0.001).

In sensitivity analyses addressing diagnostic heterogeneity, intertrochanteric BMD remained independently associated with stem size discrepancy after adding diagnosis to the primary multivariable model (adjusted β = −0.428, 95% CI − 0.561 to − 0.295, *p* < 0.001) and after restricting the analysis to the osteonecrosis subgroup (*n* = 315; β = −0.382, 95% CI − 0.560 to − 0.205, *p* < 0.001). In a linear mixed-effects model with a random intercept for patient, the effect was essentially unchanged, confirming that treating both hips as independent observations did not materially affect the results (β = −0.429, 95% CI − 0.567 to − 0.291, *p* < 0.001).

A discrepancy of ≥ + 3 sizes occurred in 28 hips (5.5%), and a discrepancy of ≤ − 3 sizes occurred in 18 hips (3.5%). ROC analysis of the intertrochanteric T-score discriminated these clinically significant discrepancies: a cutoff of − 0.9 for ≥ + 3 sizes (AUC = 0.782, 95% CI 0.693–0.865; sensitivity 82.1%; specificity 63.5%) and 0.2 for ≤ − 3 sizes (AUC = 0.825, 95% CI 0.705–0.913; sensitivity 83.3%; specificity 77.2%) (Fig. 3b, c; Table 5).

**Table 5:** Output of receiver operating characteristic (ROC) curve for discriminating stem size discrepancy (≥ 3 or ≤ − 3 sizes) using bone mineral density (T-score, intertrochanteric)

Group	Factor	AUC	95% CI	Optimal threshold	Sensitivity	Specificity
Stem size discrepancy ≥ 3	BMD (T-score, intertrochanteric)	0.782	0.693 to 0.865	-0.9	82.1%	63.5%
Stem size discrepancy ≤ − 3	BMD (T-score, intertrochanteric)	0.825	0.705 to 0.913	0.2	83.3%	77.2%

*AUC* Area under the curve, *CI* confidence interval, *BMD* bone mineral density

## Discussion

In this study, we investigated the factors associated with the accuracy of preoperative templating in cementless THA, with particular emphasis on bone quality. No significant factors were associated with discrepancies in acetabular cup size. However, age, weight, and intertrochanteric BMD were identified as independently associated with femoral stem size discrepancy. Among these, intertrochanteric BMD showed the strongest association. Patients with lower intertrochanteric BMD were more likely to require stems larger than those templated, whereas those with higher BMD tended to receive smaller stems. Although intertrochanteric BMD was selected a priori and showed the strongest univariate association with stem size discrepancy, it was highly correlated with total-hip and trochanteric BMD. Therefore, the present findings should not be interpreted as proving an anatomically exclusive effect of the intertrochanteric region. Rather, intertrochanteric BMD should be viewed as a clinically accessible marker of proximal femoral bone quantity, particularly in the metaphyseal region relevant to broaching and press-fit fixation.

The principal clinical utility of these findings is to flag, before surgery, the minority of patients in whom the implanted stem is likely to deviate substantially from the templated size, so that an adequate range of implants and instruments is prepared and intraoperative delay or implant unavailability is avoided. In our cohort, reconstruction targets were generally met (mean postoperative LLD 1.1 mm, none > 10 mm; mean offset difference 1.5 mm), indicating that the observed deviations did not compromise reconstruction.

ROC analysis identified threshold values that may help surgeons anticipate substantial deviations from the templated stem size and prepare an appropriate implant size range preoperatively. An intertrochanteric T-score below − 0.9 was associated with an increased likelihood of requiring a stem ≥ 3 sizes larger than templated, whereas a T-score above 0.2 was associated with a discrepancy of ≤ − 3 sizes. Because these thresholds were derived from a single retrospective cohort with a small number of outcome events (28 hips [5.5%] with a discrepancy of ≥ + 3 sizes and 18 hips [3.5%] with ≤ − 3 sizes), the resulting estimates are inherently unstable. They should therefore be regarded as hypothesis-generating rather than clinically actionable decision limits, and external validation in independent cohorts is required before any routine clinical application.

The Dorr classification is commonly used to assess proximal femoral bone quality. Previous studies have reported no significant differences in fixation stability of the Taperloc stem across Dorr types [23, 24]. Consistent with these findings, our results showed no meaningful differences in templating accuracy between Dorr classifications. Moreover, Mevorach et al., despite using a different stem design, similarly found no significant association between Dorr type and templating accuracy [25]. Although the Dorr classification reflects proximal femoral cortical bone morphology, our results suggest it may not adequately capture the aspects of bone quality most strongly associated with templating accuracy.

Although the Taperloc Microplasty is a tapered-wedge stem that relies primarily on mediolateral metaphyseal fit and proximal cortical engagement, cancellous and cortical contributions are not mutually exclusive. Previous biomechanical and RSA (radiostereometric analysis) studies have linked intertrochanteric cancellous bone quality and volumetric BMD to femoral stem migration in cementless THA [13, 26]. In particular, Moritz et al. reported that the quality of intertrochanteric cancellous bone predicted femoral stem RSA migration [26], and Aro et al. reported that low intertrochanteric volumetric BMD was associated with increased stem migration in postmenopausal women undergoing THA [13]. Although our study used DXA-derived areal BMD rather than volumetric measurements or direct assessment of cancellous bone quality, intertrochanteric BMD may serve as a clinically accessible surrogate for metaphyseal trabecular bone characteristics relevant to broaching and press-fit. During broaching, intertrochanteric trabecular bone may be compacted against the endosteal cortex, and the quality of this trabecular bed may influence how readily stable press-fit is achieved and, consequently, the final broach and stem size selected intraoperatively. However, trabecular compaction and intraoperative fixation strength were not directly measured in the present study; therefore, this proposed mechanism remains hypothetical, and our retrospective data support an association rather than causality.

Age, another significant factor identified in our study, may be associated with changes in bone quality, including alterations in trabecular microarchitecture and increased cortical porosity [27–29]. With aging, bone undergoes not only a reduction in bone mass but also deterioration in its material and structural properties, which are critical determinants of bone strength. These changes may compromise the ability of trabecular bone to provide adequate support during press-fit fixation, thereby contributing to discrepancies in stem sizing.

Holzer et al. also identified overweight status as a significant factor affecting the accuracy of digital templating, which aligns with our findings [9]. This may be due to magnification errors caused by incorrect positioning of the radiopaque calibration bar during imaging. Although we standardized bar placement regardless of body weight, variability in patient body habitus may have affected radiographic magnification and, consequently, templating accuracy.

This study had some limitations. First, it was a retrospective, single-center analysis in which the G7 acetabular component and Taperloc Microplasty femoral stem were used. The use of a homogeneous implant and standardized surgical technique reduced implant-related confounding when evaluating factors associated with templating accuracy. However, the findings may not be generalizable to other implant designs. In particular, the magnitude of the BMD effect and the derived thresholds may not apply to stems with different fixation philosophies, such as distally fixed extensively coated, fully hydroxyapatite-coated, modular, or short metaphyseal designs. A multicenter, multi-implant study is needed to validate the broader applicability of intertrochanteric BMD as a templating marker. In addition, no external validation cohort was available; the ROC-derived thresholds were therefore neither internally cross-validated nor externally validated, and this absence of validation itself represents a limitation of the present study.

Second, we used DXA-derived BMD rather than volumetric measurements. DXA-derived areal BMD reflects bone quantity rather than the full concept of bone quality, which also encompasses geometric, structural, and material properties; therefore, our measurements characterize regional bone quantity [30]. Although DXA has lower spatial resolution than volumetric imaging modalities, it is more accessible and widely implemented in clinical practice, which may enhance its practical utility and clinical relevance. Moreover, because all BMD measurements were obtained using a standardized protocol, relative comparisons between patients remain reliable, and this limitation is unlikely to affect the observed associations.

Third, preoperative templating was performed by orthopedic residents and clinical fellows with varying levels of experience. A dedicated intraobserver and interobserver reliability analysis was not performed in the present cohort; however, the same PACS-based on-screen templating method using calibrated radiographs and acetate overlays has previously been evaluated for accuracy and reliability at our institution [20]. All templating results were reviewed and, when necessary, revised by the operating surgeon, which likely reduced interobserver variability. Previous studies have also highlighted the role of surgeon experience in improving templating accuracy [9, 31]. Therefore, although some variability may exist, its impact was likely minimized and is unlikely to have substantially affected the overall findings.

Finally, although intertrochanteric BMD showed the strongest association among the variables examined, the multivariable model explained only a modest proportion of the variance (R² = 0.226), indicating that BMD is only one of several factors associated with intraoperative stem sizing and that most variability remains unexplained. Unmeasured factors—including quantitative proximal femoral geometry, such as the canal flare index and canal-to-calcar ratio, trabecular microarchitecture not captured by areal BMD, and intraoperative tactile feedback during broaching—are likely to contribute to stem size selection. Although all procedures were performed by a single experienced surgeon under standardized conditions, the assessment of bone texture and press-fit resistance remains inherently subjective and cannot be quantitatively calibrated.

Although Mevorach et al. examined the influence of femoral bone quality, assessed by the Dorr classification, on cementless THA templating, to our knowledge no prior study has examined the association between quantitative DXA-derived regional BMD—particularly intertrochanteric BMD—and templating accuracy [25]. Although the potential benefits of BMD-informed templating were not directly evaluated in the present study, we believe that our findings offer new and clinically meaningful insights that may improve preoperative planning and implant selection in cementless THA. Further prospective studies are needed to determine whether BMD-informed templating improves operative efficiency, inventory utilization, or clinical outcomes, and whether stem size deviation affects initial stability or complications.

## Conclusions

Among the factors examined, intertrochanteric BMD was the factor most strongly associated with femoral stem size discrepancy, although it accounted for only part of the variability. Preoperative assessment of intertrochanteric BMD may help anticipate substantial deviations in stem sizing and optimize implant preparation, pending external validation across additional implant designs and centers.

## Supplementary Information

Additional file 1. Supplementary Table S1. Correlation analysis (Spearman’s rho) for factors associated with stem size discrepancy.

## Data Availability

The datasets used and/or analysed during the current study are available from the corresponding author on reasonable request.

## Abbreviations

THA: Total hip arthroplasty
BMD: Bone mineral density
LLD: Leg length discrepancy
BMI: Body mass index
DXA: Dual-energy X-ray absorptiometry
PACS: Picture archiving and communication system
AP: Anteroposterior
PGY: Postgraduate year
ROC: Receiver operating characteristic
AUC: Area under the curve
SD: Standard deviation
SE: Standard error
VIF: Variance inflation factor
ONFH: Osteonecrosis of the femoral head
OA: Osteoarthritis
FNF: Femoral neck fracture
SIF: Subchondral insufficiency fracture
AS: Ankylosing spondylitis
