# Supplementary material for: Impact of intertrochanteric bone mineral density on the accuracy of preoperative templating in cementless total hip arthroplasty: a retrospective cohort study of 510 hips
Source: BMC Musculoskelet Disord. 2026 Aug 4;27:937. doi: 10.1186/s12891-026-10301-5 (PMC13721713; doi:10.1186/s12891-026-10301-5)
Supplement: Supplementary file 1 [file 12891_2026_10301_MOESM1_ESM.docx]

**Supplementary Table S1.** Correlation analysis for factors associated with stem size discrepancy.

| Spearman-Rho correlation coefficient (*P-*value) | Age (year) | Gender | Height (cm) | Weigh (kg) | BMI (kg/m^2^) | BMD  (femur neck) | BMD  (trochanter) | BMD  (intertrochanter) | BMD  (total hip) | Dorr type | Stem size discrepancy |
| --- | --- | --- | --- | --- | --- | --- | --- | --- | --- | --- | --- |
| Age (years) | 1 (-) | -0.271 (<0.001) | -0.473 (<0.001) | -0.345 (<0.001) | -0.056 (0.208) | -0.309  (<0.001) | -0.039  (0.374) | -0.247  (<0.001) | -0.209 (<0.001) | 0.332 (<0.001) | 0.265 (<0.001) |
| Gender |  | 1 (-) | 0.770 (<0.001) | 0.505 (<0.001) | 0.054 (0.222) | 0.237 (<0.001) | 0.035 (0.432) | 0.323 (<0.001) | 0.273 (<0.001) | -0.228 (<0.001) | -0.206 (<0.001) |
| Height (cm) |  |  | 1 (-) | 0.622 (<0.001) | 0.012 (0.784) | 0.382 (<0.001) | 0.135 (0.002) | 0.323 (<0.001) | 0.273 (<0.001) | -0.240 (<0.001) | -0.271 (<0.001) |
| Weigh (kg) |  |  |  | 1 (-) | 0.759 (<0.001) | 0.474 (<0.001) | 0.348 (<0.001) | 0.495 (<0.001) | 0.474 (<0.001) | -0.305 (<0.001) | -0.323 (<0.001) |
| BMI (kg/m^2^) |  |  |  |  | 1 (-) | 0.301 (<0.001) | 0.320 (<0.001) | 0.373 (<0.001) | 0.379 (<0.001) | -0.215 (<0.001) | -0.191 (<0.001) |
| BMD (femur neck) |  |  |  |  |  | 1 (-) | 0.572 (<0.001) | 0.651 (<0.001) | 0.729 (<0.001) | -0.358 (<0.001) | -0.329 (<0.001) |
| BMD  (trochanteric) |  |  |  |  |  |  | 1 (-) | 0.804 (<0.001) | 0.871 (<0.001) | -0.442 (<0.001) | -0.370 (<0.001) |
| BMD  (intertrochanter) |  |  |  |  |  |  |  | 1 (-) | 0.962 (<0.001) | -0.579 (<0.001) | -0.426 (<0.001) |
| BMD (total hip) |  |  |  |  |  |  |  |  | 1 (-) | -0.543 (<0.001) | -0.415 (<0.001) |
| Dorr type |  |  |  |  |  |  |  |  |  | 1 (-) | 0.290 (<0.001) |
| Stem size discrepancy |  |  |  |  |  |  |  |  |  |  | 1 (-) |

BMI, body mass index; BMD, bone mineral density.
